# Testing least cost path (LCP) models for travel time and kilocalorie expenditure: Implications for landscape genomics

**DOI:** 10.1371/journal.pone.0239387

**Published:** 2020-09-22

**Authors:** Kyle M. Gowen, Timothy S. de Smet

**Affiliations:** 1 Department of Anthropology, Binghamton University, Binghamton, NY, United States of America; 2 Laboratory of Evolutionary Anthropology and Health, Binghamton University, Binghamton, NY, United States of America; 3 Director, Geophysics and Remote Sensing Laboratory, Binghamton University, Binghamton, NY, United States of America; 4 Department of Geological Sciences and Environmental Studies, Binghamton University, Binghamton, NY, United States of America; University of Wisconsin Madison, UNITED STATES

## Abstract

Least Cost Path (LCP) analysis allows a user to define a cost parameter through which cost of movement can be assessed using Geographical Information Systems (GIS). These analyses are commonly used to construct theoretical movement through a landscape, which has been useful for creating hypotheses concerning prehistoric archaeology and landscape genomics. However, LCP analysis is commonly employed without testing the generated LCP(s), complicating its usefulness as a methodological tool. This paper proposes a model for analyzing movement in ArcGIS by using topography data to calculate slope. This slope data is then then used to calculate LCPs based on travel time and kilocalorie expenditure. LCPs were constructed in the Nature Preserve at Binghamton University, a 182-acre area that consists of wetland and mountainous terrain, and a Fitbit® Surge activity monitor was used to test the accuracy of the model’s predictions. Paired sample t-tests show a lack of significant difference between calculated and walked time in our analysis (p = .953), suggesting that our model can estimate travel time between two points based solely on slope of the region. Paired sample t-tests also show a lack of significant difference between calculated and observed kilocalorie expenditure (p = .930), suggesting that our model is also capable of estimating kilocalorie expenditure associated with movement between two points. Finally, paired sample t-tests confirm that straight line distances do not reflect real movement through a terrain (p = .009), highlighting the need for alternate measures of movement when analyzing the effects of local landscape on movement. Our current model shows strength in its estimations of travel time and kilocalorie expenditure based on topography of a region–future iterations of the model need to establish the statistical similarity between our model’s estimations and recorded values for walking time and kilocalorie expenditure.

## Introduction

This paper proposes a model for analyzing movement through a region based on topography using ArcMap, a program in the ArcGIS suite. Analyses focused on movement through complex regions have been useful in the fields of landscape genomics and archaeology for understanding how groups interact in the presence of complex geography that limits said interaction. Our model uses topography data to calculate slopes throughout a region, which are then used to calculate least cost paths (LCPs) based on time spent traveling and kilocalorie expenditure based on modified equations related to theoretical movement and energy expenditure [1–3]. We used the Nature Preserve at Binghamton University, a 182-acre natural research and recreation area that consists of wetland and mountainous terrain, as a location to construct these LCPs due to the difficulty and complexity of the terrain. Using a Fitbit® Surge activity monitor, we were able to test the distances, times, and kilocalorie expenditures calculated by our model by walking one of the established paths in the Preserve. Our methodology found no significant difference between its calculation of walked time vs. actual walked time for the seven paths included in the model (p = .953), indicating that our model is capable of estimating walking time between two points if the slope of the region is known. Our model also shows a lack of significant difference in its kilocalorie expenditure calculations (p = .930), indicating that it can also estimate kilocalorie expenditure based on the weight of the individual moving through the landscape. Finally, our model found significant difference between straight line distances and the walked trails included in the study (p = .009), indicating that straight line distances are limited in their usage as an analytical tool when considering movement and interaction through a landscape.

## Background

Over the past several decades, the development of landscape imaging and processing technology, most notably the development of Geographical Information Systems (GIS), has allowed for a variety of geographical analyses that were unheard of before the development of this technology. GIS is a multifaceted tool that can be used for a variety of different purposes and has allowed researchers to include and analyze elements of the natural world as they relate to research. Of particular note in the past several decades is the development of the field of landscape genomics [4]. Before the development of GIS and its application to population genetics, straight line distances between populations were commonly used as a metric through which to analyze genetic similarity of different groups. However, researchers were quick to recognize that the landscape of a particular region has a pronounced effect on the ability of two groups to interact and comingle [5]. Researchers pointed out that straight line distances were not an accurate representation of the geospatial reality in which biological organisms find themselves. A desire to understand the finer elements of population interaction and intermingling necessitates a consideration of the constraints that landscape places on biological organisms.

A consideration of the effect of landscape on both population interaction and movement can be accomplished in a number of ways, most notably through the construction of Least Cost Paths (LCPs). GIS is capable of producing LCPs through a complex algorithm in which the user defines for the system what parameter is being analyzed to assess cost of movement through said parameter. In analyzing landscape, the topography of the region of interest is commonly used as the cost parameter, as movement is heavily impeded when moving across areas that are heavily sloped. However, topography of the region is not the only cost parameter that can be utilized–research into maritime travel in different regions of the world have focused on using wind speeds as a cost parameter [6] or using distance to a coastal trading hub as a cost parameter [7]. Regardless of the defined parameter, GIS produces an LCP that represents the so called “easiest” route of movement through the parameter between two defined points. The “ease” of travel depends on the cost parameter defined.

The construction of LCPs allows for a more nuanced understanding of movement through a particular region, which can be used for a variety of means. In landscape genomics, the construction of LCPs is used as a more accurate representation of straight line distances between two population. LCPs take into account the effects of the landscape on the biological organisms of interest, and research has shown that constructing LCPs as a baseline for comparison of genetic distances is more viable than simply using straight line distances [8–10]. LCP construction has been used in archaeology to assess movement through a landscape both historically and prehistorically. LCP analysis has been used to identify potential site locations in relation to Holocene migration [7], determining potential movement of archaeological tool materials from distant source quarries [11,12], and has been used to analyze potential movement of prehistoric peoples through diverse landscapes [13]. LCP analysis is a useful tool in both biological and archaeological science due to its multifaceted application, as any parameter can be defined as a cost for GIS to analyze potential movement through.

One of the pitfalls of LCP analysis is that is considers movement through only a single parameter–in constructing a LCP, the cost parameter is the only thing considered. In the case of topographical analysis, GIS will calculate a LCP based solely on ease of travel through the region of interest in relation to slope. However, this can produce a much longer distance in comparison to the straight line distance between two points and does not accurately reflect real movement, as movement is not restricted solely to low slope areas. Another pitfall of LCP analysis is that the constructed paths, while representing “easy” travel, are difficult to test. While GIS can identify potential pathways of movement through a region, archaeological and historical data is needed to confirm if these constructed pathways were really traveled and utilized by the people of interest.

This project initially started as part of a landscape genomics project in which we analyzed the effects of kuru, a neurodegenerative prion disease, on the genetic diversity in the highlands of Papua New Guinea in the middle of the 20^th^ century [9]. While straight line distances were originally being used as a baseline through which to analyze the genetic similarities of different linguistic and cultural groups in the region, it was recognized that movement through the eastern highlands of Papua New Guinea (PNG) was impeded by the presence of the Wanevinti and Kratke mountain ranges [14,15]. Using GIS, we constructed LCPs throughout the region based on slope using topographical data to assess movement through the mountainous regions of the highlands. However, the construction of LCPs based on slope was limiting. Reports from Carleton Gajdusek’s initial expedition into the region revealed that movement through the highlands was based both on time of travel, not ease of slope, and culturally defined markers, such as friendly and related villages to the people traveling with the research team [16,17]. As a response to this, we constructed LCPs utilizing an assessment of travel time as the cost parameter by using Tobler’s Hiking Function to assess movement speed in relation to slope [1]. These LCPs provided an estimation of the travel time between two villages in the highlands, a useful tool for comparing genetic distances, as the analysis revealed that several villages were more than a full walking day away. A final consideration of interest for our research team was the amount of energy that would be expended by walking from one village to another, measured in kilocalories. By modifying equations in the literature, [2,3], we calculated LCPs between villages that minimized the amount of energy, measured in kilocalories, providing a third metric through which to assess genetic similarity and interaction between villages.

This initial analysis showed that our LCPs based on walking time and kilocalorie expenditure were more statistically useful as a baseline for genetic distance comparisons than simple straight line distances [9]. However, as mentioned earlier, the major pitfall of this research was that we were still unable to test the accuracy of our constructed paths, as Gajdusek’s research team did not adequately map their movement through the PNG Highlands at the time of sample collection.

This paper aims to provide a working model for LCP construction based on ease of slope, time, and kilocalories spent; furthermore, this paper provides a methodology for testing the accuracy of the constructed LCPs. The Nature Preserve at Binghamton University, a 182 acre of undeveloped land attached to the University for ecological training and outdoor recreation, provides a useful test area for our LCP constructions. The Nature Preserve extends into the southern portion of Broome County, New York and consists of 20 acres of wetland and approximately 11 miles of trails that include an approximate 500 feet increase in elevation. While not as rigorous as the highlands of PNG, the complex topography of the region allows for similar LCP construction. With LCPs constructed, we tested the model by walking the constructed paths while wearing a Fitbit Surge®, an activity monitor that includes a built in GPS, a 3-axis accelerometer, an altimeter, and can calculate calories burned based on weight, allowing for direct comparisons to our three constructed LCPs.

## Methods

### Field methods: Model location and sites

**Fig 1:**
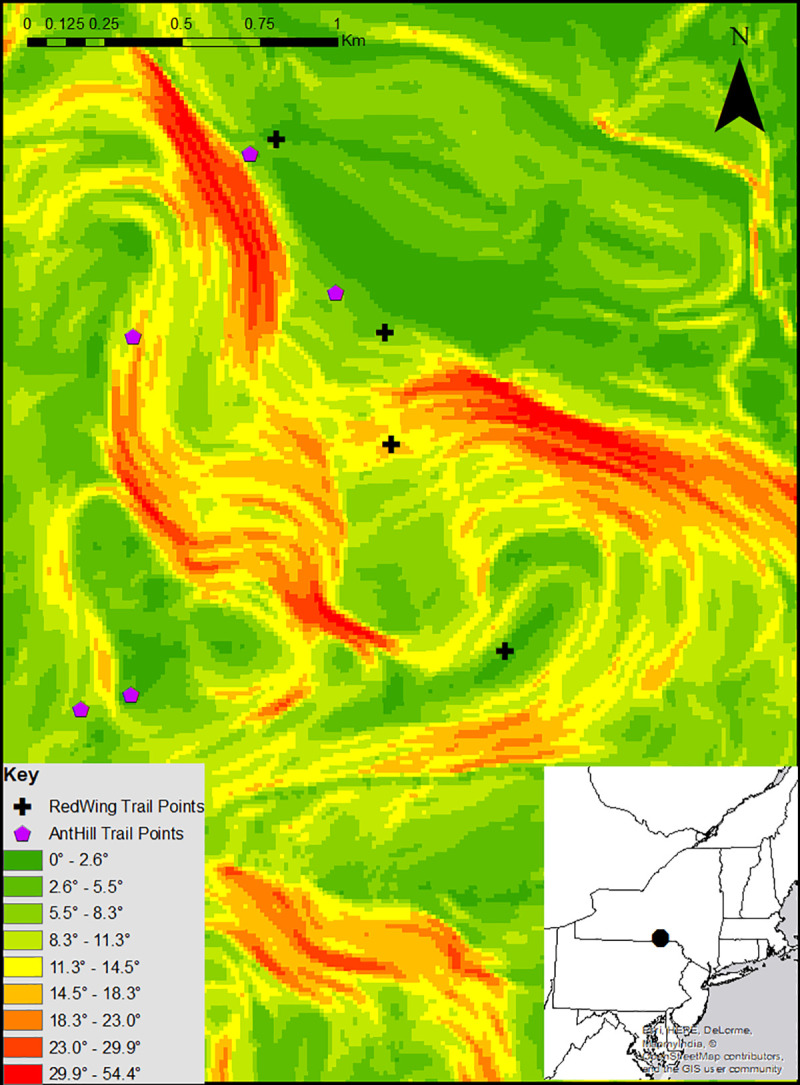
The Ant Hill and Red Wing trails in the nature preserve at Binghamton University. The trails in the Nature Preserve extend for almost 11 miles. The AH points are represented by purple pentagons (AH1 being the most northern point) and the RW points are represented by black crosses (RW1 being the most northern point).

The Nature Preserve at Binghamton University was established in 1969 and consists of 182 total acres with 20 acres of wetlands. The Nature Preserve consists of almost 11 miles of trail that extend throughout the 182 acres. For this analysis, we used ArcMap 10.6, a software program produced by Esri® as part of the overall ArcGIS program, to construct our LCPs. We decided to focus our analysis on two separate trails in the Nature Preserve, the Red Wing Trail (RW) and the Ant Hill Trail (AH)—these two trails are the two longest continuous trails in the Preserve and possess the largest changes in elevation across the trails. The AH Trail consists of five points, while the RW trail consists of four points, with AH1 and RW1 representing the most northern points of the trail and AH5 and RW4 representing the most southern points of the trail. A visualization of the seven points, overlaid the slope of the region, can be found in Fig 1.

### ARCGIS methodology: LCP construction

Topography data, in.dem format, for the Nature Preserve, which includes most of the southern half of Broome County, NY, was obtained from the Cornell University Geospatial Information Repository (CUGIR), in 10m by 10m resolution. This topography data was used to calculate slope throughout the region using ArcMap’s Slope function, as seen in Fig 1. To account for the presence of the marshland, which is impassible except for a foot bridge, GoogleEarthPro® was used to construct polygons that represent the extent of the marshland on both sides of the bridge. These polygons were then used to eliminate that region of the slope data from analysis while allowing the space that the bridge occupies to remain in the analysis. Using ArcMap 10.6, we used the beginning of the each trail as our initial source point–we then constructed LCPs in a sequential manner for both sets of trails (AH1-AH2, RW1-RW2, AH2-AH3, RW2-RW3, etc.)

The next step in the analysis was to rebuild LCPs with time as the cost parameter, which required a modification of Tobler’s Hiking Function [1]. Using the Raster Calculator tool, we used the following modified version of Tobler’s equation to convert the slope data, in degrees, into friction surface (FS) data:

$$T=\frac{\frac{R}{1000}}{{6e}^{-3.5|\tan\left(\frac{D\pi }{180}\right)+0.05|}}$$

where R is the spatial resolution of the DEM data in meters, D is the slope of the data in degrees, and T is time in hours.

This FS data allows us to reuse the Cost Distance and Cost Path tool, this time using the FS data as the cost parameter. The Cost Distance and Cost Path tool rebuilds the LCPs in units of hours traveled rather than meters traveled. By optimizing time, the constructed LCP is slightly different from the LCP in which slope was the cost parameter, as the Cost Distance and Cost Path tools calculate a path that is willing to take a more inclined path if it minimizes the amount of time traveled.

The final set of LCPs to be constructed utilized calories spent as the cost parameter, which required two sets of calculations. To begin, the slope data, in degrees, needed to be converted into velocity data, which could identify movement across a cell based on the slope of said cell. The equation, modified in [13] from [2], calculates this velocity in meters per second, and is as follows:

$$V={6e}^{-3.5|\tan\left(\frac{D\pi }{180}\right)+0.05|}(C)$$

where *V* is velocity, also called walking speed, modified by multiplying the result by a conversion factor C (1000/3600, or 0.277778) to convert kilometers per hour to meters per second [18].

With the expected velocity of the region calculated, the next step in the process was to calculate the metabolic rate of travel (MRT) in watts (Joule/s) per second. The equation for this is as follows:

$$MRT=1.5W+2({\frac{L}{W})}^{2}+\eta (W+L)(1.5V^{2}+0.35V(\tan\left(\frac{D\pi }{180}\right)100))$$

In which *W* is an individual’s weight in kilograms, *L* is the external load in kilograms, V is the velocity calculated in the prior step, and η is a terrain coefficient. For our purposes, we constructed the model around a the lead author (Gowen), in which the weight of the individual was calculated at 113.4 kg (250 lbs) and the load weight was calculated at 7 kg (approximately 15 lbs). The terrain variable is a coefficient representing the effort of crossing the terrain based upon land cover classifications. For the purposes of this model, the Nature Preserve was modeled as a mixed forest environment with a terrain coefficient of 1.5.

Lastly, the MRT in watts per second was converted into kilocalories per second via multiplication by a conversion factor (1 W = 0.000239 kcal/s) and divided by the previously calculated velocity in meters per second to calculate the number of kilocalories expended during travel in kilocalories per meter. This calculation is as follows:

$$kcal=\frac{(0.000239)MRT}{V}$$

With movement through the region calculated in kilocalories per meter, the Cost Distance tool can be used to calculate the calories spent moving from one point to another while the Cost Path tool can calculate the lay of this energetically efficient movement.

### Field methods: Testing LCPs

By using established walking trails in the Nature Preserve, our model allows us to assess the accuracy of each LCPs by traversing the path between two points and measuring distance, time, and kilocalorie expenditure. Just as Gajdusek noted that movement through the highlands of PNG was limited by various factors, movement through the Nature Preserve is similarly constrained by the topography of the region of Broome County and the hiking trails themselves, as several of the trails are surrounded by dense brush that is difficult to move through. Using a Fitbit® Surge, which includes an altimeter, a speedometer, a heartrate tracker and a GPS, we walked from the start of the Redwing trail to RW2, collecting distance, time, and kilocalorie data, along with mapping the trail itself. After reaching RW2, we ended the data collection and began a new data collection session to build sequential walking paths that reflected the way in which we constructed our model and walked to RW3. Upon reaching RW3, we ended the data collection and began a new session and completed the walk to the end of the trail. We repeated this sequential data collection with the Ant Hill Trail on a separate day. In this way, the data collection periods reflect the sequential pattern in our model. The Fitbit Surge® provides three different types of data, one for each of our LCPs, that allows us to assess the how accurate our calculated LCPs are to the recorded values.

## Results

### Straight line distances

The first step in generating our model was to calculate slope data for the region. Slope throughout the southern half of Broome County, NY ranges from 0°, represented as a deep green color, to 54.39°, represented as a deep red color, as shown in Fig 1.The entire span of the.dem file is approximately 13.64 km north-south by 10.30 km west-east. This.dem data extends from the township of Johnson City on the northern border of the Susquehanna River to the NY/PA border in the southernmost aspect of Broome County. Before beginning LCP construction, we used ArcMap to measure the straight line distances sequentially between points of the RW and AH trail to reflect the sequential nature of our model. These straight-line distances can be found in Table 1 –the mapped points of the RW and AH Trail can be seen in Fig 1.

**Table 1:** Straight line distances between the points of analysis.

	Distance (m)	Distance (km)
RW1 to RW2	517.2	.5172
RW2 to RW3	267.7	.267
RW3 to RW4	562.0	.562
Total of RW Combined Segments	1346.9	1.347
RW1 to RW4	1324.73	1.325
AH1 to AH2	389.3	0.3893
AH2 to AH3	496.3	0.4963
AH3 to AH4	853.9	0.8539
AH4 to AH5	124.9	0.1249
Total of AH Combined Segments	1864.3	1.864
AH1 to AH5	1387.6	1.3876

The total of the RW Trail segments measure approximately 1.35 km long, with the second segment between RW2 and RW3 as the shortest segment at 0.268 km and the segment from RW3 to RW4 as the longest segment at .562 km. The AH Trail is slightly longer than the RW Trail, measuring 1.86 km in total, with the segment between AH4 to AH5 being the shortest at 0.125km and the segment between AH3 to AH4 being the longest at 0.85km. For comparison, the straight line distance between the start and end of the trail was also included–in both cases, the combined sequential straight line distances are longer than straight line distance, highlighting the importance of considering topography when analyzing distance traveled.

### Calculated time LCPs

**Fig 2:**
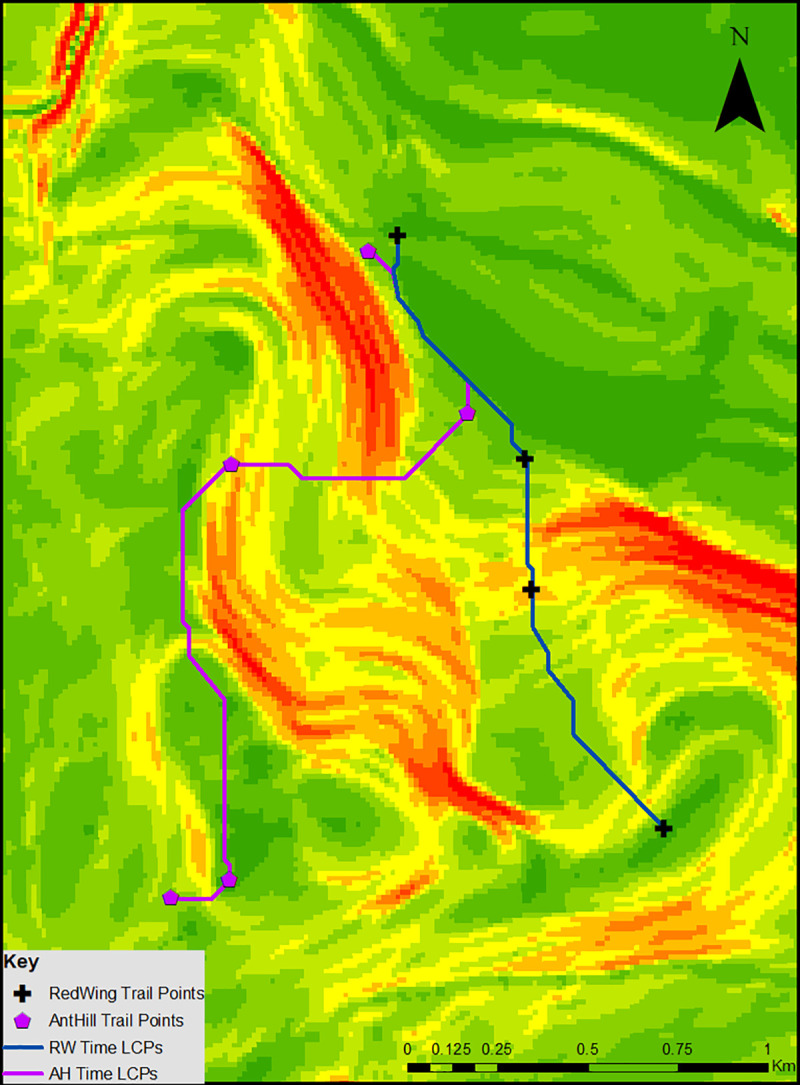
Time LCPs for the RW and AH trails.

As described in Methods, we used ArcMap to calculate FS data from the slope, allowing us to assess how slope impedes travel time. Using this FS data as a new cost parameter, we were able to calculate cost of movement in amount of time traveled in hours. The travel time between the points in each trail can be found in Table 2, with the paths mapped in Fig 2. Travel time between the points of analysis in whole hours and minutes can be found in the first two columns. Travel time, converted into minute:second format, can be found in the third column.

**Table 2:** Time LCPs between the four points of analysis.

	Time (hr)	Time (min)	Time (m:s)
RW1 to RW2	.143	8.56	8:34
RW2 to RW3	.112	6.73	6:44
RW3 to RW4	.207	12.44	12:26
Total of RW Combined Segments	.462	27.73	27:44
RW1 to RW4	.455	27.33	27:20
AH1 to AH2	.110	6.58	6:35
AH2 to AH3	.230	13.81	13:49
AH3 to AH4	.249	14.97	14:58
AH4 to AH5	.042	2.53	2:32
Total of AH Combined Segments	0.631	37.86	37:52
AH1 to AH5	0.558	33.45	33:27

The Nature Preserve at Binghamton University, while occupying 182 acres, represents only a small portion of the total of the southern half of Broome County. Both the AH and RW Trail represent a total straight line distance of less than two kilometers, so travel time for the entirety of the trail is less than an hour. As shown in Table 2, the model estimates that walking the entirety of the RW Trail from start to finish in a sequential manner should take just under 30 minutes (27:44), while walking the entirety of the AH Trail in the same manner should take just over 30 minutes (37:52). For comparison and in conjunction with the measured straight-line distances, estimated walking time was also calculated from the beginning of the trail to the end. In both cases, moving from the start of each trail towards the end in a non-sequential manner should take less time than doing so sequentially (27:20 vs. 27:44 for the RW Trail, 33:27 vs. 37:52 for the AH Trail.)

### Calculated kilocalorie expenditure LCPs

**Fig 3:**
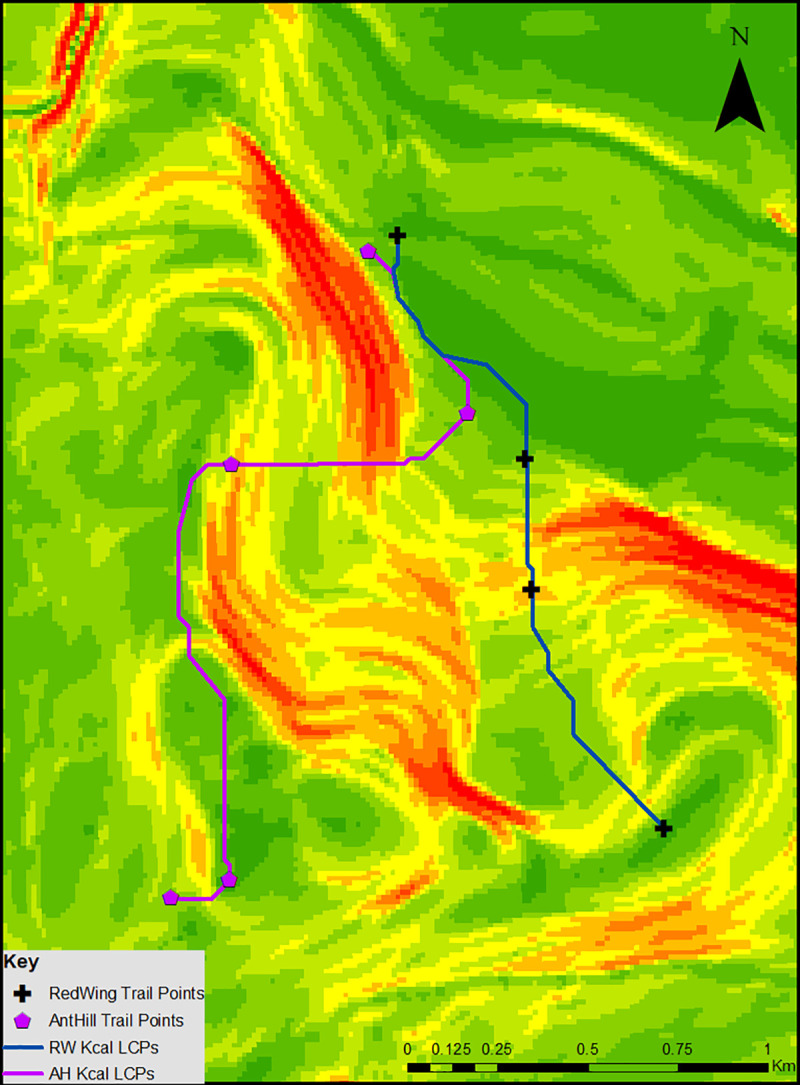
Kilocalorie LCPs overlaid the slope layer.

The final step in constructing the model was to calculate LCPs based on minimizing calorie expenditure, in which we used the slope of the region to calculate expected velocity (EV) and metabolic rate of travel (MRT) using the equations outlined in Methods. Kilocalorie expenditure for each segment of both trails can be found in Table 3, with the constructed paths visualized in Fig 3.

**Table 3:** Calculated kilocalorie expenditure between the points of analysis.

	Calculated Kilocalorie Expenditure
RW1 to RW2	104.55
RW2 to RW3	99.77
RW3 to RW4	178.93
Total of RW Combined Segments	383.25
RW1 to RW4	379.95
AH1 to AH2	83.97
AH2 to AH3	202.45
AH3 to AH4	189.88
AH4 to AH5	35.12
Total of AH Combined Segments	511.42
AH1 to AH5	447.71

Reflecting similar trends in the Time LCPs, the kilocalorie expenditures for each segment of both trails reflect the amount of time it takes to traverse the segment. In our model, moving from RW2 to RW3 and AH4 to AH5 expends the least amount of calories due to their shortness. However, as seen in Fig 3, crossing between the points in both trails requires crossing areas of high slope, resulting in high kilocalorie expenditure in certain segments, most notably RW3 to RW4 (178.93) and AH2 to AH3 (202.45). Following trends seen in both straight line distance and the time LCPs, moving sequentially between the points of the trail expends more calories than being able to move from the start of the trail to the end, as seen in Table 3.

### Walked data and comparisons to the model

**Fig 4:**
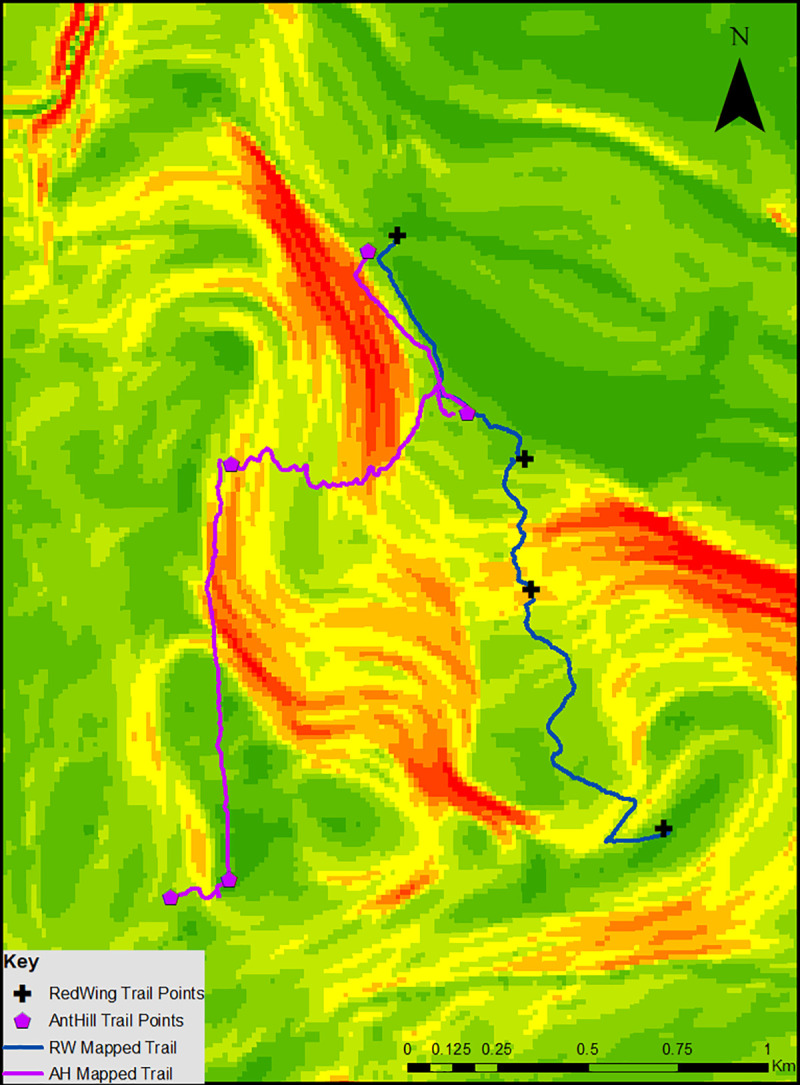
Sequential walking paths, as mapped using a Fitbit® Surge, overlaid the slope layer.

Using a Fitbit® Surge, we walked the trails calculated in our model—these sequential paths are visualized in Fig 4 and summarized in Table 4.

**Table 4:** Distance and time data collected by Fitbit® Surge.

	Distance (miles)	Distance (km)	Time (minutes:seconds)	Kilocalories burned
RW1 to RW2	0.43	0.692	8:57	111
RW2 to RW3	0.28	0.451	6:19	72
RW3 to RW4	0.58	0.933	12:50	133
Total of RW Combined Segments	1.29	2.08	28:06	316
AH1 to AH2	0.30	0.483	6:56	123
AH2 to AH3	0.55	0.885	13:33	227
AH3 to AH4	0.59	0.950	12:36	209
AH4 to AH5	0.11	0.177	2:15	27
Total of AH Combined Segments	1.55	2.494	35:20	586

The Fitbit® Surge collected distance data in miles rather than kilometers, so the distance for each walking path was converted into kilometers using a conversion rate of 1 mile = 1.60934 kilometers, rounded to three decimals. Time data was collected in minutes:seconds format and kilocalorie expenditure data was calculated by the Fitbit® based on a calculated BMR by using the Mifflin-St. Jeor equation [19,20]. The Fibit® Surge shows a total walked distance of just over two kilometers for both trails. In the RW Trail, the path between RW3 to RW4 is the longest segment of the walk, while in the AH Trail, the path between AH3 and AH4 is the longest segment of the trail. The total time for both walks rests around the 30 minute mark, with the RW Trail taking less time to traverse than the AH Trail, most likely due a difference in total distance of each trail (2.08km vs. 2.49km respectively). The kilocalorie expenditure calculated by the Fitbit® Surge shows that a total of 316 kilocalories were burned during the 28 minute RW Trail walk, while the AH Trail walk burned a total of 586 kilocalories. Kilocalorie expenditure does not necessarily reflect distance, as show in Table 4, as the path between AH2 to AH3 expends more kilocalories (227) than the path between AH3 to AH4 (209), despite the fact that the latter is longer (0.89km vs. 0.95km respectively).

For ease of comparison, straight line distance and walked distance, calculated time and walked time, and calculated kilocalorie expenditure and recorded kilocalorie expenditure for the sequential paths can be found in Table 5.

**Table 5:** Calculated vs. walked distance, time, kilocalorie expenditure.

	Straight line distance (km)	Walked distance (km)	Calculated Time (m:s)	Walked Time (m:s)	Calculated kilocalorie	Kilocalories burned
RW1 to RW2	0.5172	0.692	8:34	8:57	104.55	111
RW2 to RW3	0.2677	0.451	6:44	6:19	99.77	72
RW3 to RW4	0.5620	0.933	12:26	12:50	178.93	133
Total of RW Combined Segments	1.347	2.08	27:44	28:06	383.25	316
AH1 to AH2	0.389	0.483	6:35	6:56	83.97	123
AH2 to AH3	0.496	0.885	13:49	13:33	202.45	227
AH3 to AH4	0.854	0.950	14:58	12:36	189.88	209
AH4 to AH5	0.125	0.177	2:32	2:15	35.12	27
Total of AH Combined Segments	1.864	2.494	37:52	35:20	511.42	586

This table shows that the total walked distance for both trails is higher than the straight line distance (2.08km vs. 1.35km and 2.49km vs. 1.86km), which we originally predicted due to the topography of the region and the presence of the marshland. The calculated total walk time, at 27:44 for the RW Trail and 37:52 for the AH Trail, is similar to the actual walked total time at 28:06 and 35:20 respectively. For the RW Trail, the calculated walking time for each segment is within half a minute of the actual observed walking time. For the AH Trail, the only segment that has more than a 30s difference between calculated walking time and observed walking time is the path from AH3 to AH4 (14:58 vs. 12:36 respectively). For the individual segments of each trail, the calculated kilocalorie expenditure falls within 50 kilocalories of the observed kilocalorie expenditure, as seen in Table 5. For the combination of the segments of each trail, the calculated kilocalorie expenditure is higher than the observed expenditure for the RW Trail (383 vs. 316) but lower than the observed expenditure for the AH Trail (511 vs. 586). This most likely reflect differences in the topography of the region, as moving along each trail represents two different experiences in regards to the slope that is being traversed, as seen in Figs 3 and 4.

Based on these comparisons, we conducted paired sample t-tests to determine whether are calculations were significantly different than the observed values for each set of comparisons (distance, walked time, kilocalorie expenditure.) Before conducting this comparison, we removed the total of the RW Combined Segments and the total of the AH Combined Segments from Table 5, as these variables represent sums of the previous segments and break the assumptions necessary to perform the paired sample t-test. With these two variables removed, we imported Table 5 into IBM SPSS version 20 and ran paired sample t-tests for the three sets of comparisons–the results of these tests are summarized in Table 6.

**Table 6:** Paired sample t-tests comparing modeled distance, time, and kilocalorie expenditure to those recorded by the Fitbit® Surge.

	t	Df	p
Distance	3.804	6	.009
Time	-.061	6	.953
Kilocalorie	.092	6	.930

Starting with the distance comparisons, these tests confirm that the straight line distances between the points of analysis are significantly different than the distance of the walked paths (t = 3.804, p = .009). Pointing to the strength of our model, these paired t-tests do not find significant difference between our calculated walking times and the observed walking times (t = -.061, p = .953) and between our calculated kilocalorie expenditure and the observed kilocalorie expenditure (t = .092, p = .930). This suggests that our model’s calculations of both walking time and kilocalorie expenditure are useful for estimating approximately how long it would take to move through an area and how much energy said movement would take if the slope of the area is known.

**Fig 5:**
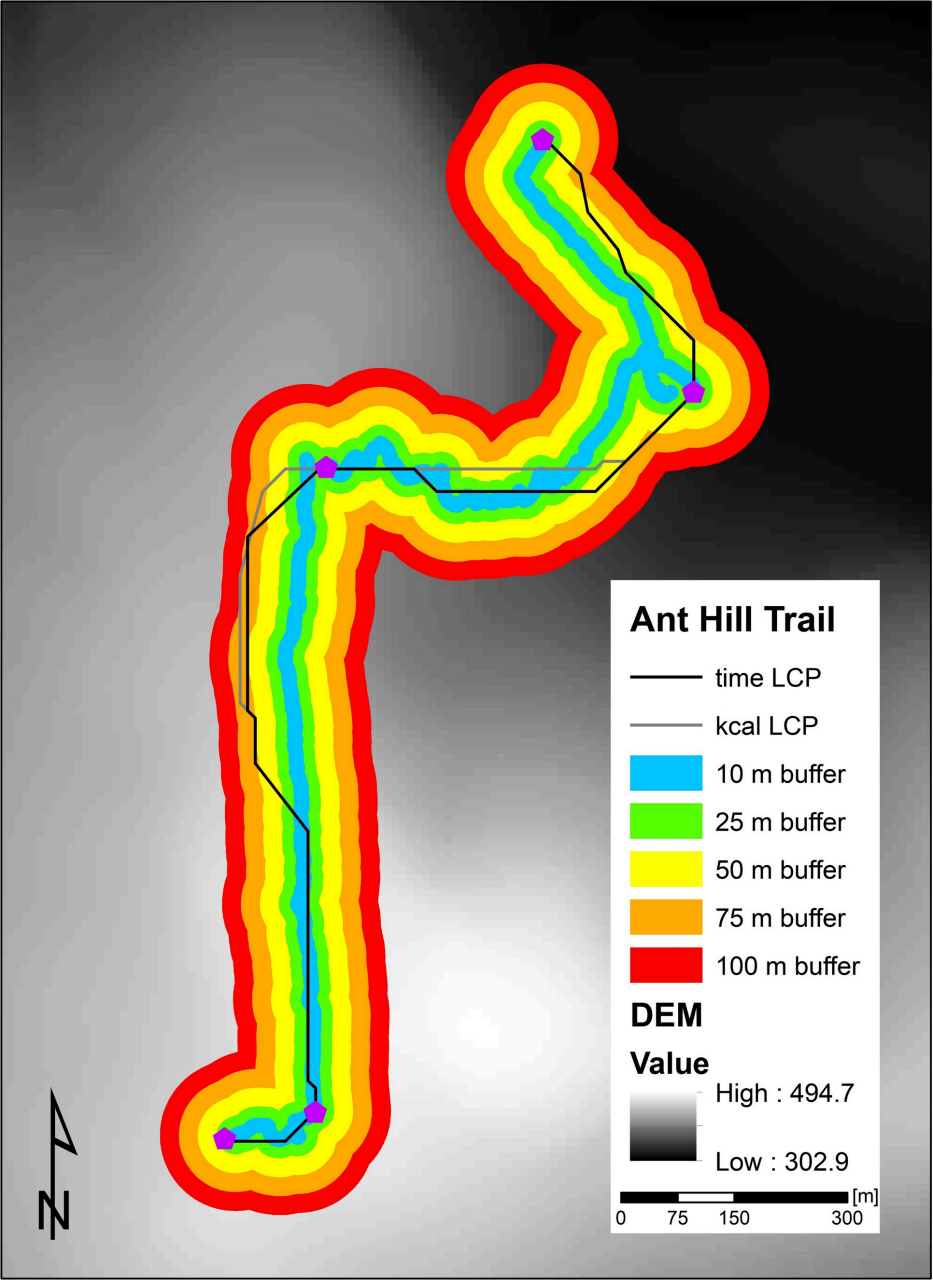
Ant Hill Trail buffers at 10, 25, 50, 75, and 100m about the actual paths and LCPs used to determine correlation factors.

The second quantitative method used to compare the actual trails to the LCPs was through an assessment of correlation factors [21]. The correlation factor is defined as the percentage that the modelled LCPs overlap with the buffered trails, as mapped with the Fitbit® Surge. The geometry of the LCP is calculated as Length in m (Length_*LCP*_) and the actual trails are buffered at 10, 25, 50, 75, and 100 m on both sides of the trail. The LCP was then intersected with the buffers. The geometry of this intersection between the LCP and the buffers around the path is calculated as Length in m (*Length*_*intersect*_). The correlation factor is calculated by taking *Length*_*intersect*_*/ Length*_*LCP*_−this correlation factor is represented as the percentage of overlap for the various buffer lengths around the recorded walked trails described previously (10/25/75/100m). This quantitative metric is a useful tool to determine the correlation between actual trails and modeled LCPs. The assessment of correlation factors for the Ant Hill Trail is displayed in Fig 5.

## Discussion

The model we have built allows for the generation of LCPs in ArcMap based on distance, time, and kilocalorie expenditure. These constructed LCPs can be analyzed in a qualitative fashion by comparing the constructed paths to the actual mapped path, which can be seen by comparing Figs 2, 3 and 4. The actual walked paths can also be compared quantitatively to the model’s predicted paths by calculating correlation factors, as described above. Using a fitness tracker, such as the Fitbit® Surge, allows us to test these LCP constructions quantitatively by comparing the model’s predictions with the observed values, in this case, observed distance, walked time, and kilocalorie expenditure. The combination of both qualitative and quantitative comparisons allows us to assess the strength of the model and determine if our model is capable of accurately estimating different assessments of movement through a landscape.

### Straight line vs. walked paths

The first comparison that our model allows us to make is between the straight-line distances involved in each trail and the actual walked paths between the points in each trail. While the straight line distances are not included in a figure, Fig 4 clearly shows that the paths in the Nature Preserve do not follow straight lines, as the topography and flora of the Preserve dictate possible movement. As an example, following a straight line between RW1 and RW2 would cross through one of the thickest parts of the marsh, as indicated by the dark green areas representing a slope of 0°, which is incredibly difficult, if not outright impossible. Quantitatively, the Fitbit® shows that the walked paths are longer than the straight line distances, as seen in Table 5, and do not follow straight lines. Paired sample t-tests, as shown in Table 6, confirm that a significant difference exists between the straight line distances between each point of the trail and the actual walked distance between said points (p = .009). This finding agrees with the academic literature that straight line distances do not accurately reflect the amount of distance traveled between two points when topography is considered [11–13].

### Calculated vs. actual walked time

Qualitatively, the paths that the model constructs to minimize walked time, as shown in Fig 2, between each point in the trail are straight edged and minimize the amount of time spent in high slope regions (as indicated by yellow and red colors). This is due to the reduction in walking speed associated with increased slopes following Tobler’s Hiking Function (see Methods). When comparing Figs 2–4, we see that the recorded paths are much more jagged and less direct than the paths that the time LCPs follow. Most notably, the path between AH2 to AH3 and the path between RW3 and RW4 are much more convoluted than their time LCP. This is due to two factors: the topography of the region and the vegetation encountered on the trail itself. In the case of AH2 to AH3, this segment of the trail moves through a significantly sloped area (as visualized by the yellow and red color). To make this path easier, the trail is designed with a series of switchbacks that make traveling the segment easier, if albeit more convoluted than the hypothetical path that minimizes time traveled between them. In addition to this, the vegetation in the Nature Preserve can also serve as a barrier to movement. In the segment between RW3 and RW4, the trail traverses dense tree and brush coverage–while it is possible to move through this vegetation, it is difficult. In the case of our model, while the model accounts for topography by including slope in its calculation, the model does not include an assessment of vegetation coverage that could influence movement decisions. An understanding of vegetation could be useful in the model to further understand how landscape affects movement between two points in a particular region.

Despite the differences between the calculated path and the actual trails, Table 6 shows that paired sample t-tests fail to find significant difference between our model’s estimation of walking time and the observed walking time as measured through a Fitbit® Surge (p = .953).

As mentioned earlier, the methodology described has been used successfully in landscape genomic research as a metric for similarity between groups of PNG highlanders [9]. We were unable to test the accuracy and validity of those LCP constructions due to the lack of recorded information about walking paths between remote villages and the remoteness of the villages included in the analysis. Through the application and subsequent testing of our methodology in the Nature Preserve, we have demonstrated the strength of our model due to lack of evidence of a difference between our estimates of walking time and actual observed walking time. While future iterations of the model need to account for various landscape factors (water, vegetation, etc.), this research confirms that our model can accurately estimate walking time between two points based solely on slope, making it a useful methodological tool.

### Calculated vs. actual kilocalorie expenditure

When comparing the constructed LCPs based on walked time and kilocalorie expenditure, four of the seven paths are copies of the time LCPs, as both calculations factor slope into their path construction. The three kilocalorie LCPs that differ from their corresponding time LCPs are the segment between AH2 to AH3, AH3 to AH4, and the segment between RW1 to RW2. In the case of the former, the discrepancy between the two LCPs is due to the model’s desire to minimize the amount of time spent in high slope areas, as high slope areas are more calorically costly to cross than low slope areas. The segment between AH2 to AH3 moves directly through a high slope area to cross it as quickly as possible while the segment between AH3 to AH4 quickly moves out of a high slope area to a low sloped one. In both cases, the model is attempting to minimize kilocalorie expenditure of the path. In the case of the segment between RW1 and RW2, the model minimizes kilocalorie expenditure by staying in the low slope area around the marshland (as indicated by the deep green color in Figs 1–4), while the corresponding time LCP is willing to cross through slightly higher sloped areas to minimize time spent traveling. In all three cases highlighted here, the discrepancies between the time LCPs and kilocalorie LCPs is due to the cost they are trying to minimize–the time LCPs are willing to move through high sloped areas if it minimizes travel time while the kilocalorie LCPs attempt to avoid these areas as they represent high kilocalorie expenditure.

As highlighted in Table 5, for each of the seven segments, our model’s estimated kilocalorie expenditure is within 50 kilocalories of the observed values as recorded by the Fitbit® Surge. Paired sample t-tests, as shown in Table 6, indicate that there is no significant difference between our model’s estimations and the observed values for the seven segments included in the analysis (p = .930). In similar fashion to our time LCPs, this points to the strength of our model–we found no evidence for a statistical difference between our model’s estimation of kilocalorie expenditure and the observed values, which makes it a useful tool for estimating the energy cost associated with movement across a landscape. Further iterations of the model could include an assessment of how height and age of the hypothetical traveler affect kilocalorie expenditure. The Mifflin-St. Jeor equations that the Fitbit® Surge relies on include height, weight, and age in their calculation of kilocalorie expenditure [19,20] while our model only considers weight. Including height and age as variables could allow our model to estimate kilocalorie expenditure more accurately than when weight and load weight are considered as the only variables.

### Calculated vs. actual paths

**Fig 6:**
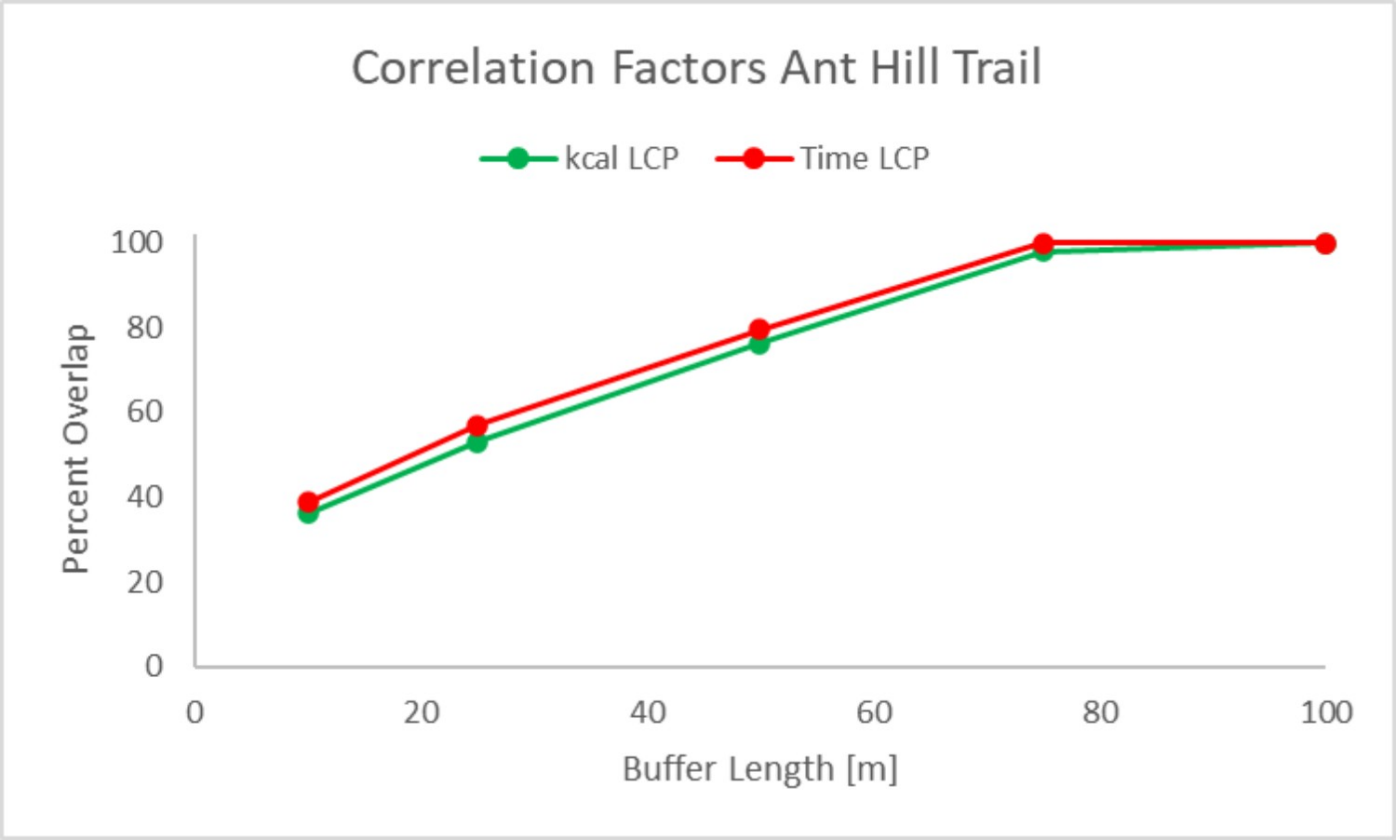
Correlation factors to determine correlation between actual and kcal and time LCPs on the Ant Hill Trail.

Correlation factors are a useful quantitative metric to compare the discrepancies between the actual trails and LCP models geometries. The AH and RW Trails were naturally produced by human decisions over a several year time period in the late 1960s and early 1970s and have remained the same for around 50 years. As shown in Fig 5 and quantified in Fig 6, the model’s LCPs for both time and kilocalorie expenditure closely resemble the actual walked path, as the model’s paths deviate by less than 75m from the walked path—above this 75m buffer around the walked path, the walked path and the LCPs possess a correlation factor of 100%. This high correlation between the model’s paths and the walked paths is also notable because the 75m buffer zone around the walked path represents less than 4% of the path’s total length, which indicates that one of the strengths of our model is its ability to approximate the walked path within a 75m zone.

**Fig 7:**
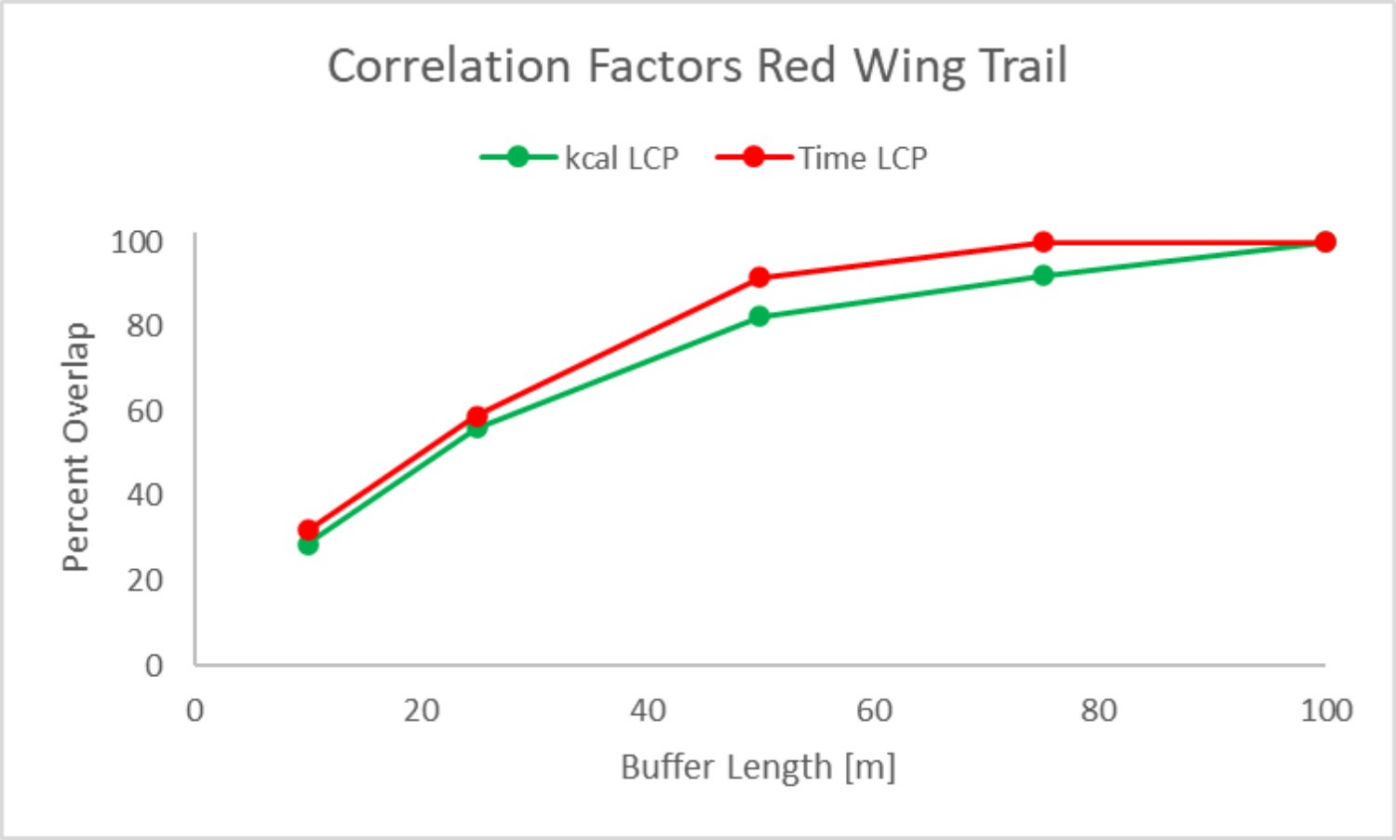
Correlation factors to determine correlation between actual and kcal and time LCPs on the Red Wing Trail.

Research utilizing this approach has commonly produced low interaction overlaps, such as a study in the Chaco Canyon area using 500 m buffers resulted in an intersection overlap as low as 25% [21]. In comparison, our high interaction overlaps at smaller buffer zones, such as approximately 45% overlap at a 20m buffer as seen in Fig 6, points to the strength of our model, as our model deviates less than 75m away from the recorded trails. The RW LCPs more closely mirrored the actual trail. At 10 m the predicted path for AH Trail are more accurate than RW Trail, but for all other buffers RW is more accurate, as shown in Fig 7.

Neither AH or RW modeled paths deviate by > 100 m for the actual trails. The time LCP is more accurate than the kcal LCP at both AH and RW Trails–this indicates that the time LCP algorithm may better fit the geometry of actual paths. This finding is interesting and should be considered in future iterations and expansions of this research. The greater accuracy of the time LCP may also indicate that the people who made these paths value time more than calories, which is potentially true given the paths were created in the 1960s in the United States; testing this on prehistoric paths could potentially provide another meaningful evaluation of this in the future.

### Strengths and limitations

Our model possesses three major strengths. Firstly, in line with other LCP methodologies, paired sample t-tests confirms that straight line distances do not reflect observed movement through a region (p = .009), even within the small scale used in our model’s constructions. Secondly, for six out of the seven segments, our model is capable of estimating walking time within half a minute of the observed values and paired sample t-tests found no significant difference between our seven estimates and the observed walking times for the seven segments (p = .953). As mentioned previously, while LCP analysis is used in a variety of biological and archaeological research, the predictions from these analyses are rarely ever tested due to a variety of complicating factors. Our model’s estimation of walking time is unique in that we did not find evidence for a difference between our model’s estimations and the observed walking times, validating its use as a methodological tool in other research that relies on or benefits from LCP analysis. The last strength of our model, in tandem with the estimations of walking time, is that our model is capable of estimating kilocalorie expenditure within 50 kilocalories of the observed values and paired sample t-tests found no significant difference between our estimates and the recorded kilocalorie expenditures (p = .930). These estimations provide an understanding of the hypothetical energy expenditure that it takes to move between two points in space, which provides a metric for comparison and analysis in landscape genomics [9]. In summary, the strength of our model lies in our ability to validate and test the LCPs in question–having tested estimations of walking time and kilocalorie cost provides a more nuanced understanding of movement across a landscape then simply applying LCP analysis uncritically.

While our model provides a useful technique for establishing metrics for comparisons that could be useful to landscape genomic and other archaeological analyses, the model possesses limitations that future iterations of the model could improve and refine. One particularly noteworthy area of improvement for our model is developing a technique to address the presence of water. Water inherently reads as a zero-degree slope, which makes the analysis difficult as any potential water ways are very low slope and are favored by the model when conducting a slope-based analysis. Future iterations of the model will need to develop a cost parameter that combines the slope of the region with its waterways, marking these waterways as high cost (rather than low cost) to accommodate their presence. Another potential area of improvement is that the individual moving across the landscape is not included in the time calculation. Both the model and the Fitbit® include a consideration of the individual in its kilocalorie calculations in the form of weight and height, weight, and age respectively. However, in regards to the time LCP, the only variable included in the calculation is slope of the region–no consideration is given to the individual. While the model is inherently an optimizing system, future iterations of the model could assess how physical fitness affects travel time across a landscape. Another area in which the model could improve its estimations, particularly in regards to the actual paths it constructs across a landscape, is a more nuanced and complex understanding of the region being traveled. As mentioned earlier, vegetation throughout the Nature Preserve acts as a barrier to movement–while the model is capable of estimating travel time and kilocalorie expenditure fairly well, the mapped path does not accurately reflect the trails in the Preserve due to these complicating factors. By including more complex factors, such as vegetation coverage and considering how seasonality affects ease of movement, our model could more accurately estimate what the easiest path across a certain landscape looks like.

## Conclusion

We have constructed a model that allows for the analysis of movement through a region based on topography using ArcMap. Using the Nature Preserve at Binghamton University as a case study, we used topography data to calculate slope throughout the Preserve. Once the slope of the region was calculated, our model calculated LCPs based on minimizing travel time and kilocalorie expenditure for two separate trails that represent the greatest length and change in elevation in our case study. To assess whether our model’s estimations were accurate, we walked the two trails included in the analysis while wearing a Fitbit® Surge activity watch to collect distance, time, and kilocalorie data for the trails.

Our model revealed three things–firstly, that straight line distances do not reflect actual walked distance as confirmed through paired sample t-tests (p = .009). Secondly, paired sample t-tests reveal a lack of significant difference between the model’s estimated travel time and the recorded travel time (p = .953). Lastly, paired sample t-tests revealed no significant difference between the model’s estimated kilocalorie expenditure and the recorded expenditure (p = .930). Our analysis failed to find a difference between our model’s calculations and the recorded values for comparison–this suggests that our model’s strengths lie in it is ability to accurately estimate walked time and kilocalorie expenditure if the slope of the region is known. While the model’s strength rests in its estimations, further iterations of the model need to develop a methodology for assessing how the presence of water affects movement. Additionally, the model could benefit from a more complex understanding of how factors such as vegetation and seasonality also affect movement across a landscape. In conclusion, we have failed to find a significant difference between our model’s estimations for travel time and kilocalorie expenditure and the observed values, pointing to its utility as a methodological tool for biological and archaeological studies in which an understanding of movement between two points of interest is needed.
